# Comparative Performance of Spectral Reflectance Indices and Multivariate Modeling for Assessing Agronomic Parameters in Advanced Spring Wheat Lines Under Two Contrasting Irrigation Regimes

**DOI:** 10.3389/fpls.2019.01537

**Published:** 2019-11-28

**Authors:** Salah E. El-Hendawy, Majed Alotaibi, Nasser Al-Suhaibani, Khalid Al-Gaadi, Wael Hassan, Yaser Hassan Dewir, Mohammed Abd El-Gawad Emam, Salah Elsayed, Urs Schmidhalter

**Affiliations:** ^1^Department of Plant Production, College of Food and Agriculture Sciences, King Saud University, Riyadh, Saudi Arabia; ^2^Department of Agronomy, Faculty of Agriculture, Suez Canal University, Ismailia, Egypt; ^3^Department of Agricultural Engineering, Precision Agriculture Research Chair, College of Food and Agriculture Sciences, King Saud University, Riyadh, Saudi Arabia; ^4^Department of Agricultural Botany, Faculty of Agriculture, Suez Canal University, Ismailia, Egypt; ^5^Department of Biology, College of Science and Humanities at Quwayiah, Shaqra University, Riyadh, Saudi Arabia; ^6^Department of Horticulture, Faculty of Agriculture, Kafrelsheikh University, Kafr El Sheikh, Egypt; ^7^Evaluation of Natural Resources Department, Environmental Studies and Research Institute, University of Sadat City, Menoufia, Egypt; ^8^Department of Plant Sciences, Technische Universität München, Freising, Germany

**Keywords:** partial least squares regression, phenomics, phenotyping, proximal sensing techniques, recombinant inbred lines, stepwise multiple linear regression, wavelength band selection

## Abstract

The incorporation of nondestructive and cost-effective tools in genetic drought studies in combination with reliable indirect screening criteria that exhibit high heritability and genetic correlations will be critical for addressing the water deficit challenges of the agricultural sector under arid conditions and ensuring the success of genotype development. In this study, the proximal spectral reflectance data were exploited to assess three destructive agronomic parameters [dry weight (DW) and water content (WC) of the aboveground biomass and grain yield (GY)] in 30 recombinant F7 and F8 inbred lines (RILs) growing under full (FL) and limited (LM) irrigation regimes. The utility of different groups of spectral reflectance indices (SRIs) as an indirect assessment tool was tested based on heritability and genetic correlations. The performance of the SRIs and different models of partial least squares regression (PLSR) and stepwise multiple linear regression (SMLR) in estimating the destructive parameters was considered. Generally, all groups of SRIs, as well as different models of PLSR and SMLR, generated better estimations for destructive parameters under LM and combined FL+LM than under FL. Even though most of the SRIs exhibited a low association with destructive parameters under FL, they exhibited moderate to high genetic correlations and also had high heritability. The SRIs based on near-infrared (NIR)/visible (VIS) and NIR/NIR, especially those developed in this study, spectral band intervals extracted within VIS, red edge, and NIR spectral range, or individual effective wavelengths relevant to green, red, red edge, and middle NIR spectral region, were found to be more effective in estimating the destructive parameters under all conditions. Five models of SMLR and PLSR for each condition explained most of the variation in the three destructive parameters among genotypes. These models explained 42% to 46%, 19% to 30%, and 39% to 46% of the variation in DW, WC, and GY among genotypes under FL, 69% to 72%, 59% to 61%, and 77% to 81% under LM, and 71% to 75%, 61% to 71%, and 74% to 78% under FL+LM, respectively. Overall, these results confirmed that application of hyperspectral reflectance sensing in breeding programs is not only important for evaluating a sufficient number of genotypes in an expeditious and cost-effective manner but also could be exploited to develop indirect breeding traits that aid in accelerating the development of genotypes for application under adverse environmental conditions.

## Introduction

The agriculture sector in arid and semiarid regions utilizes the maximum amount of available water abstracted from the rivers or groundwater, accounting to an average of approximately 70% of available fresh water resources (El-Hendawy et al., 2017a). With the unprecedented competition for the limited water resources between different water-consuming sectors, the governments in these regions have issued many regulations to reduce the amount of water allocated to the agriculture sector. Therefore, one of the most important objectives to meet the challenge of water-limited supplies is to apply the most feasible strategies that ultimately maximize water productivity (Fereres and Soriano, 2007; El-Hendawy et al., 2017a). Development of new genotypes that are capable of producing high yield stability under deficit irrigation conditions by enhancing their drought tolerance is one of those strategies (Sinclair, 2011; Leufen et al., 2013; El-Hendawy et al., 2017a).

An enhanced performance of in-depth multidimensional descriptions of phenotypic parameters related to drought tolerance for a sufficient number of crossing lines is urgently required when developing drought tolerance in breeding programs (Leufen et al., 2013; El-Hendawy et al., 2015; Becker and Schmidhalter, 2017; Garriga et al., 2017). There is a need for the in-depth description of phenotypic plant traits to close the gap between plant genetics, physiology, and phenomics studies, and is also of vital importance, especially for developing genotypes with an advantageous series of phenotypes or mechanisms related to drought tolerance (Houle et al., 2010). Unfortunately, the comprehensive evaluation of plant traits using field-based plant sampling is destructive, and cost- and time-inefficient. This emphasizes the rising need for the development of phenotyping and phenomics tools and algorithms that help in obtaining a multidimensional description of the phenotypic plant traits in an expeditious and nondestructive manner. Hyperspectral canopy reflectance is one of the most recent and promising tools for achieving this objective.

The spectral signatures reflected from the plant canopy at specific wavelengths provide various types of cumulative information on the substantial and gradual changes that occur in specific plant characteristics or tolerance levels. These spectral signatures are closely associated with drought-induced changes that take place in several biochemical and biophysical plant characteristics, such as plant pigment concentrations, photosynthetic efficiency, internal leaf structures, green biomass, vegetative vigour, and plant water status (Gutierrez et al., 2010; Erdle et al., 2013; Lobos et al., 2014; Becker and Schmidhalter, 2017; Silva-Perez et al., 2018; El-Hendawy et al., 2019a; Lobos et al., 2019). Such changes in biochemical and biophysical plant characteristics, which can be related to genotypic differences and drought stress levels, can be detected through the substantial changes that tack place in the spectral signatures of the canopy measured in the visible (400–700 nm), near-infrared (700–1300 nm), and shortwave-infrared (1300–2500 nm) regions. The close association between the different plant characteristics and canopy spectral signatures indicates that the canopy spectral reflectance can thus be exploited for indirect estimation of different physiological and agronomic parameters that eventually are related to either healthy or stressed plants. However, the information of canopy spectral reflectance is not exploited until it is translated into specific simple normalized difference or ratio spectral reflectance indices (SRIs), which most of the studies have depended on SRIs for predicting plant traits of interest.

Several published SRIs have been used to successfully estimate different parameters such as aboveground biomass and water content, leaf area index, gas exchange and transpiration rates, stomatal conductance, ion and pigment contents, carbon isotope discrimination, yield components, and grain yield in several field crops under either normal or abiotic stress conditions (Erdle et al., 2013; Li et al., 2014; Lobos et al., 2014; El-Hendawy et al., 2015; Bayat et al., 2016; Becker and Schmidhalter, 2017; Garriga et al., 2017; Kawamura et al., 2018; El-Hendawy et al., 2019a; El-Hendawy et al., 2019b). For example, in diverse studies, several SRIs, which are related to plant biomass, plant water status, and plant photosynthetic efficiency, such as the green normalized difference vegetation index (GNDVI), normalized difference vegetation indices (NDVIs), SRIs related to normalized water indices (NWI-1, NWI-2, NWI-3, and NWI-4), and normalized difference moisture index (NDMI: 2200; 1100) showed significant correlation with final grain yield and explained more than 70% of yield variability under contrasting water irrigation regimes (Shanahan et al., 2001; Aparicio et al., 2002; Prasad et al., 2007; Lobos et al., 2014; Elazab et al., 2015; El-Hendawy et al., 2017a). In addition, several studies have also reported that the SRIs formulated based on NIR wavelengths such as different SRIs related to normalized water indices (NWIs), SRIs that incorporate a combination of SWIR/VIS wavelengths such as the water band index (WABI) and SWIR/NIR wavelengths such as the normalized difference water index-1640 (NDWI-1640), normalized difference moisture index (NDMI), and three-band index (SRI (860, 1640, 2130)), red edge/NIR/SWIR wavelengths such as the three-band index (SRI (690, 905, 1550)) or VIS/NIR/SWIR wavelengths such as the three-band index (SRI (974, 518, 1392) and SRI (762, 518, 1930)) were found to be effective for tracking changes in plant water status under various water treatments (Gutierrez et al., 2010; Rischbeck et al., 2014; Yao et al., 2014; Junttila et al., 2016; Elsayed et al., 2017; Rapaport et al., 2017; El-Hendawy et al., 2019a). These indicate that we can deal with different SRIs as indirect selection traits like the traditional physiological traits related to photosynthesis efficiency (photosynthesis rate, stomatal conductance, and transpiration rate) or those related to plant or leaf water status like relative water content, leaf water potential, and equivalent water thickness. Indirect selection is based on the fact that the trait employed for this selection (SRIs) and the trait used for direct selection (destructive traits) are subjected to the same pressure in a particular environment. Therefore, several studies have dealt with SRIs as indirect selection traits and their potential as an indirect selection tool has been evaluated based on the basis of its genetic correlation and heritability (Jackson, 2001; Babar et al., 2006; Gutierrez et al., 2010). Most importantly, these studies indicated that an indirect selection trait (SRIs) should have higher heritability than the direct trait (agronomic or physiological traits), and high genetic correlation with the direct trait.

However, because SRIs use only 2–3 wavebands from the full spectrum (350–2500 nm) and the spectral reflectance is strongly affected by both biochemical and biophysical characteristics of the canopy, it is difficult to construct unified SRIs to estimate measured parameters across different genetic materials and years, and contrasting growing conditions (Li et al., 2014; Kawamura et al., 2018). Furthermore, these SRIs only target the spectral information over a wide range of wavebands, while losing the critical available spectral information in specific narrow wavebands (Hansen and Schjoerring, 2003; Stellacci et al., 2016). This issue can be addressed by employing multivariate analysis techniques, which provide more flexibility in estimating the measured parameters, because they take into account the full spectrum or a wider portion of wavelengths (Herrmann et al., 2011; Hernandez et al., 2015; Garriga et al., 2017).

Stepwise multiple linear regression analysis (SMLR) is one of the multivariate analyses that can be used to extract critical wavebands associated with biochemical and biophysical properties of interest (Thenkabail et al., 2000). However, if the number of predictors (X) remarkably exceeds the number of observations (Y) (overfitting) and/or several predictors are highly correlated with each other (multicollinearity), both of which are inherent to spectral data, the MLR will fail to efficiently address both problems (Nguyen and Lee, 2006). Therefore, several studies have reported that the predictive ability of SMLR could be improved by employing only the most influential wavelengths in the final model by employing appropriate wavelength selection methods before its use (Kawamura et al., 2008; Wu et al., 2012; Li et al., 2016).

Unlike SMLR, partial least squares regression (PLSR) overcomes the problems inherent to spectral data by transforming the original predictor variables into a small number of new variables called orthogonal latent variables (OLVs) by using the characteristics of SMLR and principal component analysis (Wold et al., 2001; Balabin et al., 2007). Therefore, PLSR could be used to construct predictive models for analysing hyperspectral data and extracting the important wavebands from the full-spectrum to estimate the measured parameters, especially when hyperspectral data are analyzed across different growing conditions and genetic materials. Since the PLSR efficiently deals with the problems inherent to canopy hyperspectral reflectance, this method has been considered superior to the SRIs-based method for estimating and predicting various measured parameters such as grain yield, leaf dry weight, leaf area index, nitrogen and nutrient contents, transpiration rate, and photosynthetic capacity (Hansen et al., 2002; Cho et al., 2007; Weber et al., 2012; Li et al., 2014; Sharabian et al., 2014; Rischbeck et al., 2016; Elsayed et al., 2017; Barmeier et al., 2018).

In classical breeding programs, the final grain yield per see (GY) is often considered to be the main screening criterion for identifying the more suitable genotypes for each target environment. Because of the strong interactions between the environment and genotype for this trait, plant breeders commonly seek plant traits other than grain yield as screening criteria. However, these traits will become increasingly important as screening criteria if they demonstrate a high genetic correlation with grain yield and have high heritability values under different environmental conditions, as well as are capable of detecting high yielding lines early and efficiently from a sufficient number of crossing-lines (Babar et al., 2006; Gutierrez et al., 2010). In this sense, dry weight (DW) and water content (WC) of aboveground biomass can facilitate the prediction of grain yield at the early growth stage, of which, the former indicating the photosynthetic size of the canopy (Royo et al., 2003) and the latter indicating the amount of water available for transpiration in leaves (Sun et al., 2008).

In this study, we propose that it is possible to exploit the canopy spectral signature to expand the use of high-throughput phenotyping sensing for breeding purposes by providing plant breeders with important information to increase the chances of recognizing genotypes that are well-adapted to water shortages, creating indirect nondestructive traits that can be used as an alternative to destructive traits, and/or estimating the complex destructive traits in a rapid and cost-efficient way. Therefore, the main objectives of this study were to (1) evaluate the potential use of new and published SRIs to estimate the destructive parameters (DW, WC, and GY) for advanced breeding wheat lines under full and limited irrigation regimes; (2) assess the potential of these SRIs as an indirect nondestructive tool based on their heritability and genetic correlations for breeding purposes under both conditions; and (3) develop different SMLR and PLSR models using the effective wavelengths, different groups of SRIs, or the full spectral region (350–2500 nm) and compare their performance with those of indices that individually estimate the destructive parameters.

## Materials and Methods

### Experimental Details

Thirty recombinant F7 and F8 inbred lines (RILs) developed from a cross between the drought-susceptible genotype Sids 1 and the drought-tolerant genotype Sakha 94 were evaluated with their parents under full irrigation (FL) and limited water irrigation (LM) during 2014/2015 (F7) and 2015/2016 (F8). The seeds of the two parents were provided by the Wheat Research Center at the Agricultural Research Center, Ministry of Agriculture and Land Reclamation Giza, Egypt.

The two field experiments were conducted at the Research Station of the Food and Agriculture Sciences College, King Saud University, Riyadh, Saudi Arabia, situated at 24°25′N, 46°34′E, and 400 m above mean sea level. The temperature and precipitation during the wheat growing period (December to April) ranged between 20.2°C and 33.4°C for the maximum temperature, between 9.0°C and 20.4°C for the minimum temperature, and between 8.0 and 23.8 mm for precipitation. The soil texture at the experimental site is sandy loam with a pH of 8.2, along with soil hydraulic characteristics of 0.151 m^3^ m^-3^, 0.067 m^3^ m^-3^, and 1.51 g cm^-3^ for field water capacity, permanent wilting point, and soil bulk density, respectively.

The experiments were performed using a randomized complete block split-plot design with three replicates. The two irrigation treatments and the wheat RILs were distributed randomly in the main plots and subplots, respectively. Each subplot size was 4 m × 1.5 m (6.0 m^2^ in total area). The seeds of each RIL or parent were planted in 10-row subplots on December 5 of each season and at a seeding rate of 150 kg ha^-1^. Each subplot was fertilized at a rate of 180 kg N, 31 kg P_2_O_5_, and 60 kg K_2_O ha^-1^. Nitrogen fertilizer was applied in three equal doses at the seedling (ZS 13), middle of tillering (ZS 23), and beginning of booting growth stages (ZS 43) of Zadoks growth stages (Zadoks et al., 1974). The entire doses of P and K were applied at sowing and at the stem elongation stage, respectively. The N, P, and K fertilizers were applied as urea (46.0% N), monocalcium phosphate (15.5% P_2_O_5_), and potassium chloride (60% K_2_O), respectively.

The amount of irrigation water applied for the FL treatment was calculated based on the reference evapotranspiration (ETo, mm day^-1^) and the crop coefficient (Kc) of spring wheat. The daily meteorological data, which were collected from a weather station located 150 m from the experimental site, were applied to the FAO-56 Penman-Monteith equation given by Allen et al. (1998) to estimate ETo. The Kc values that are recommended by FAO-56 for spring wheat were adjusted based on the actual values of wind speed and relative humidity of the study area (El-Hendawy et al., 2017b). Averaged over two seasons, the cumulative amount of irrigation for the FL treatment based on the above calculation was approximately 6,000 m^3^ h^-1^. This amount of irrigation water was reduced to 50% for the LM treatment. The irrigation treatments began 2 weeks after sowing. A low-pressure surface irrigation system was used and consisted of a main water pipe that distributed water to submain hoses at each subplot. Each main water pipe was equipped with a water meter, whereas each submain hose was equipped with a manual control valve to monitor and control the amount of irrigation water delivered to each irrigation treatment.

### Measurements

Spectral data of canopy reflectance were collected at the middle anthesis growth stage (Zadoks growth stage 65) under sunny and windless conditions around midday (10:00–15:00 local time; UTC+2) (using the nonimaging portable ASD spectroradiometer; Analytical Spectral Devices Inc., Boulder, CO, USA). This sensor captures the spectral data in the range between 350 to 2,500 nm with sampling intervals of 1.4 and 2.2 nm for the spectral regions 350–1,000 nm and 1,000–2,500 nm, respectively. However, spectral data were finally interpolated automatically to 1.0 nm continuous bands. The spectral reflectance was taken at a nadir with a 25° field of view from 80 cm above the wheat canopy to cover a sufficiently large sensing area of the wheat canopy (∼23.0 cm in diameter). A Spectralon white reference panel (40 cm × 40 cm) covered with a mixture of white paint and barium sulfate (BaSO_4_) was used to calibrate the reflectance measurements. This calibration was performed prior to the measurements and every 15 min to overcome any changes in solar irradiance and atmospheric conditions during measurements. An average of five sequential measurements and 20 scans for each was recorded as the measured spectrum per subplot. The five measurements were taken for the four central rows within each subplot, excluding the first meter from both sides of each row to eliminate border effects.

After the spectral data of canopy reflectance has been collected, an area of 0.15 m^2^ from each subplot and within the scanned area (two 0.5-m consecutive rows) was cut from the ground level and its fresh weight (FW) was immediately recorded. The plant samples were cut into small pieces and oven-dried at 70°C to a constant weight and then weighed to obtain the final dry weight (DW). The aboveground dry weight per square meter (DW) was estimated based on a land-area basis using the length and width of the harvested area, whereas the water content of aboveground biomass (WC) was calculated as the ratio between the quantity of water (FW – DW) and DW and was expressed as a percentage.

When plants reached maturity, an area of 1.8 m^2^ (four 3-m consecutive rows) was harvested from each subplot, and spikes were separated and threshed. Thereafter, the final grain yield (GY) was weighed and expressed as tons ha^−1^ after the moisture content of the seeds was adjusted to approximately 14%.

### Data Analysis

Data were analyzed using XLSTAT statistical package software (vers. 2019.1, Excel Add-ins soft SARL, New York, NY, USA), while the figures were constructed using Sigma Plot (Sigma Plot 11.0). The RILs and their parents were clustered into different groups based on DW, WC, and GY simultaneously under FL and LM treatments, and by combining both treatments (FL+LM). The cluster analysis was performed using Euclidean distance and the unweighted pair-group method with arithmetic mean (UPGMA).

The spectral data of canopy reflectance collected separately from the FL and LM treatments were used to calculate different published and newly developed spectral reflectance indices (SRIs). All SRIs were selected based on their sensitivity to changes in leaf pigmentation, photosynthetic efficiency, leaf/tissue structure, leaf area index, biomass, and plant water status. The SRIs developed in this study were selected based on contour maps, which facilitated the evaluation of all possible dual wavelength combinations from binary, normalized spectral indices, and extents of hot spot regions that enabled the assessment of each trait target being studied (Elsayed et al., 2015; Stratoulias et al., 2015). The contour maps show matrices of the coefficients of determination of the relationship between the trait target and possible combinations of two individual wavelengths in the full spectral region (350–2500 nm) (**Figure S1**). The different contour maps were drawn using the R package “lattice” from the software R statistics version 3.0.2 (R Foundation for Statistical Computing, 2013). Based on hot spots in different contour maps, 26 single wavelengths (440, 480, 550, 557, 570, 580, 590, 622, 700, 710, 738, 748, 751, 760, 780, 790, 812, 850, 900, 970, 1,250, 1,450, 1,500, 1,650, 2,058, and 2,100 nm) were extracted and used to construct different SRIs. These SRIs and published SRIs are shown in **Figure 2**. The published SRIs used in the present study included a normalized difference vegetation index (NDVI, Mistele and Schmidhalter, 2008), an optimized soil-adjusted vegetation index (OSAVI, Rondeaux et al., 1996), a modified triangular vegetation index (MTVI, Haboudane et al., 2004), an enhanced vegetation index (EVI, Jiang et al., 2008), a normalized water index-2 (NWI-2, Babar et al., 2006), SRI_1100,351,1392_ (El-Hendawy et al., 2019a), and a normalized difference moisture index (NDMI, Lozano et al., 2007).

Determination coefficients (R^2^) of the linear relationship between each SRI and the measured parameters were used to evaluate the performance of SRIs individually for estimating the measured parameters.

Genotypic correlations between the measured parameters and the SRIs were estimated under individual irrigation treatments and the combined dual-treatment using the following equation described by Singh and Chaudhary (1977):

$$\mathrm{rg}=\left({\mathrm{Cov}}_{\mathrm{xy}}\right)/\surd \left({\mathrm{Var}}_{x}\times {\mathrm{Var}}_{y}\right)$$

where Cov and Var indicate components of covariance and variance between trait x (measured parameter) and trait y (SRI), respectively.

The broad-sense heritability calculated in this study measures the proportion of the phenotypic variance that is the result of genetic effects (Falconer, 1989). Therefore, the different variance components associated with the phenotypic (σ^2^_P_) and genotypic (σ^2^_G_) variance were estimated (**Table S1**) to calculate the broad-sense heritability (H^2^) for each parameter and SRI under FL, LM, and FL+LM across two years using the following equation:

$$H^{2}=\sigma_{G}^{2}/\sigma_{P}^{2}$$

The phenotypic variance (σ^2^_P_) was calculated using the following equation:

$$\sigma_{P}^{2}=\sigma_{G}^{2}+\left(\frac{\sigma_{\mathrm{GE}}^{2}}{e}\right)+\left(\frac{\sigma_{e}^{2}}{re}\right)$$

where σ^2^*_G_* is the genetic variance, σ^2^*_GE_* is the genotype × environment interaction, σ^2^*_e_* is the residual variance, e is the number of environment, and r is the number of replications.

PLSR and SMLR analyses were used to extract the effective spectral band intervals and wavelengths that most significantly contributed to the estimation of the measured parameters. To avoid underfitting or overfitting of the spectral data, PLSR analysis was applied under the optimal number of latent variables (ONLVs) using leave-one-out cross-validation (LOOCV). The ONLVs maximized the covariance between predictors (Y, spectral data) and response variables (X, measured parameters). According to the criterion of covariance maximization, the important latent variables explained most of the variance of X and Y. The ONLVs were selected according to the cumulative (cum) values of Q^2^ cum, R^2^Y cum, and R^2^X cum (**Figure S2**). Q^2^ cum was the cumulative variation of the Y and X variables predicted by the extracted ONLVs of the model and used to describe the predictive quality of the model. R^2^Y cum and R^2^X cum were the cumulative sum of squares (SS) of the variation of the Y or X variables, respectively, and explained by the extracted ONLVs of the model and utilized for describing the goodness of the fit. In general, models with Q^2^cum > 0.5 and R^2^Y cum and R^2^X cum close to 1.0 are most acceptable as the best models (Eastment and Krzanowski, 1984).

After the ONLVs were identified, the effective spectral band intervals that most significantly contributed to the measured parameter estimation under each irrigation treatment and the combined treatments were extracted based on variable importance in the projection (VIP) and loading weights of PLSR analysis simultaneously for the ONLVs. The sensitive band intervals were extracted when their VIP value was greater than 1.0, coinciding with a high absolute loading weight (Wold et al., 2001). These sensitive band intervals were further applied to SMLR analysis as independent variables to extract the exact influential wavelength contributing to the target parameter estimation, which is not possible in PLSR analysis.

Models for target parameter estimation were constructed using PLSR and SMLR. The SMLR models were constructed based on the most influential wavelengths selected for each parameter under each treatment and the group of SRIs that covered VIS/VIS, NIR/VIS, NIR/NIR, SWIR/VIS, SWIR/SWIR, or SWIR/NIR, whereas the PLSR models were constructed based on all influential wavelengths, all SRIs groups or the full spectral region (350–2500 nm). The predictive performance of all models was estimated using the coefficient of determination (R^2^) and the root mean square error (RMSE). The performance ability of the cross validation of PLSR based on the full spectral region was assessed by R^2^, RMSE, and relative error (RE, %) in both calibration and validation data sets. Twenty-five percent of data sets were applied for validation, while the remaining data sets were included in the training set.

## Results

### Grouping Genotypes Under Different Irrigation Treatments

**Figure 1:**
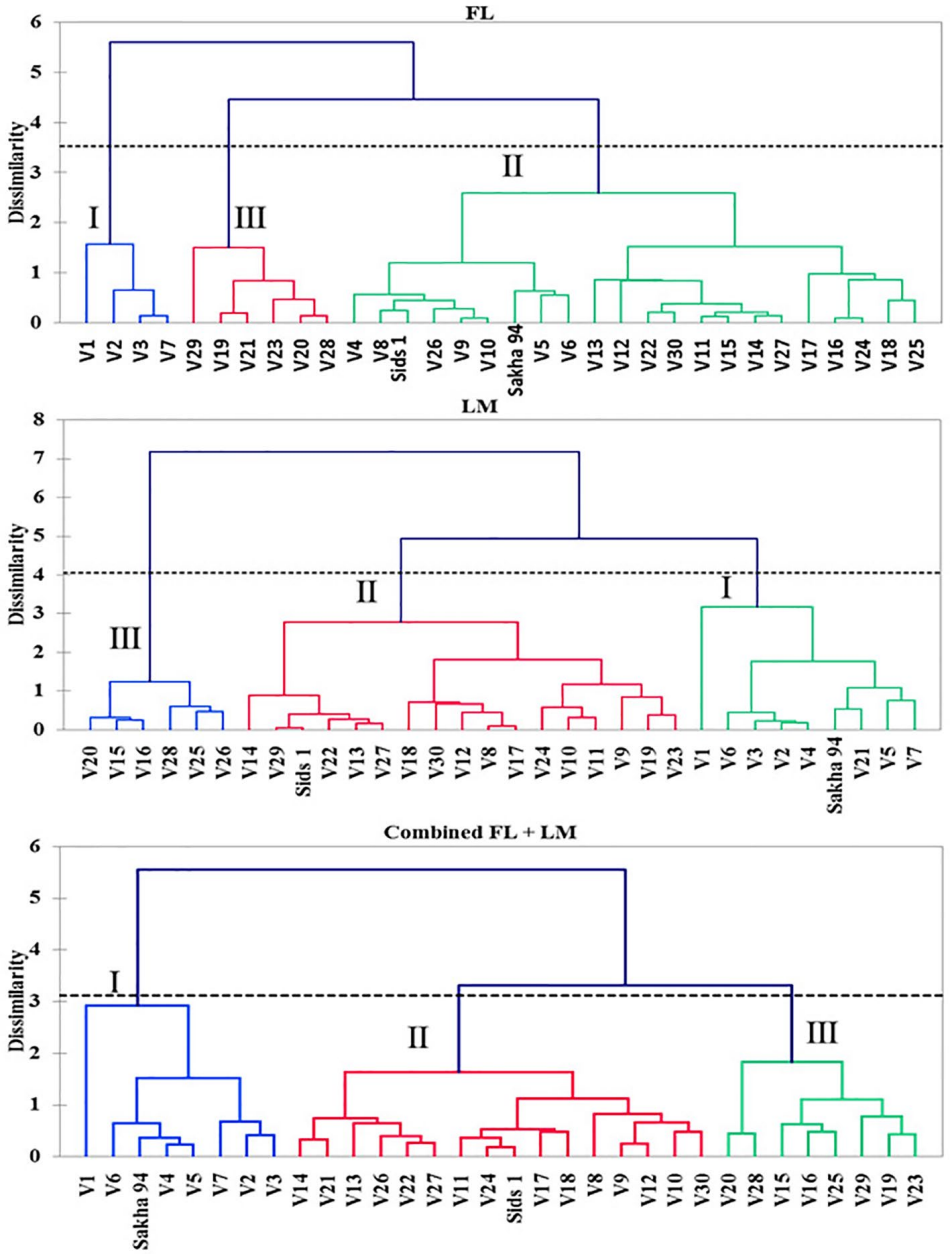
Hierarchical clusters analysis of the 32 genotypes based on measured parameters using Euclidian distance matrix and unweighted pair-group method arithmetic average (UPGMA) under full irrigation (FL), limited irrigation (LM), and the combined two treatments (combined FL+LM).

**Figure 1** shows the hierarchical cluster of the 30 recombinant inbred lines (RILs) and their two parents based on shoot dry weight per square meter (DW), water content of aboveground biomass (WC), and grain yield per hectare (GY) under full irrigation (FL), limited irrigation (LM), and the two treatments combined (FL+LM). All genotypes were grouped into three main clusters under three conditions. Even though the drought-tolerant genotype, Sakha 94, and the drought-susceptible genotype, Sids 1, were grouped together in cluster 2 under FL, they were separated in cluster 1 and cluster 2, respectively, under LM and FL+LM (**Figure 1**). Cluster 1, 2, and 3 included 4, 20, and 6 RILs under FL; 8, 16, and 6 RILs under LM; and 7, 15, and 8 RILs under FL+LM, respectively (**Figure 1**). The results in **Table 1** show that the genotypes in cluster 1 attained a higher value for the three measured parameters; the opposite was true for the genotypes in cluster 3. The mean values of DW, WC, and GY of the genotypes in cluster 2, which included most RILs and Sids 1, decreased by 17.1%, 6.8%, and 23.8% under LM and by 19.5%, 5.9%, and 18.8% under FL+LM, respectively, when compared with the mean values of the genotypes in cluster 1 (**Table 1**).

**Table 1:** Mean values ± standard deviations of shoot dry weight per square meter (DW), water content of aboveground biomass (WC), and grain yield per hectare (GY) of the three clusters group under full irrigation (FL), limited irrigation (LM) and the combined two treatments (FL+LM).

Treatment	Cluster	Number of RILS	DW (kg m^-2^)	WC (%)	GY (ton ha^-1^)
**FL**	**1**	**4 RILs**	2.71 ± 0.11	79.0 ± 1.96	8.39 ± 0.65
	**2**	**20 RILs + 2 parents**	2.10 ± 0.09	74.4 ± 2.22	6.46 ± 0.34
	**3**	**6 RILs**	1.59 ± 0.11	70.7 ± 2.84	5.30 ± 0.42
**LM**	**1**	**8 RILs + Sakha 93**	1.58 ± 0.07	68.9 ± 2.43	4.62 ± 0.12
	**2**	**16 RILs + Sids 1**	1.31 ± 0.10	64.2 ± 1.64	3.52 ± 0.41
	**3**	**6 RILs**	0.95 ± 0.11	58.8 ± 1.97	2.50 ± 0.51
**FL+LM**	**1**	**7 RILs + Sakha 93**	2.10 ± 0.13	73.5 ± 1.76	6.23 ± 0.42
	**2**	**15 RILs + Sids 1**	1.69 ± 0.14	69.2 ± 1.62	5.06 ± 0.43
	**3**	**8 RILs**	1.39 ± 0.10	65.9 ± 1.82	4.03 ± 0.37

### Relationships Between Spectral Reflectance Indices (SRIs) and Measured Parameters

**Figure 2:**
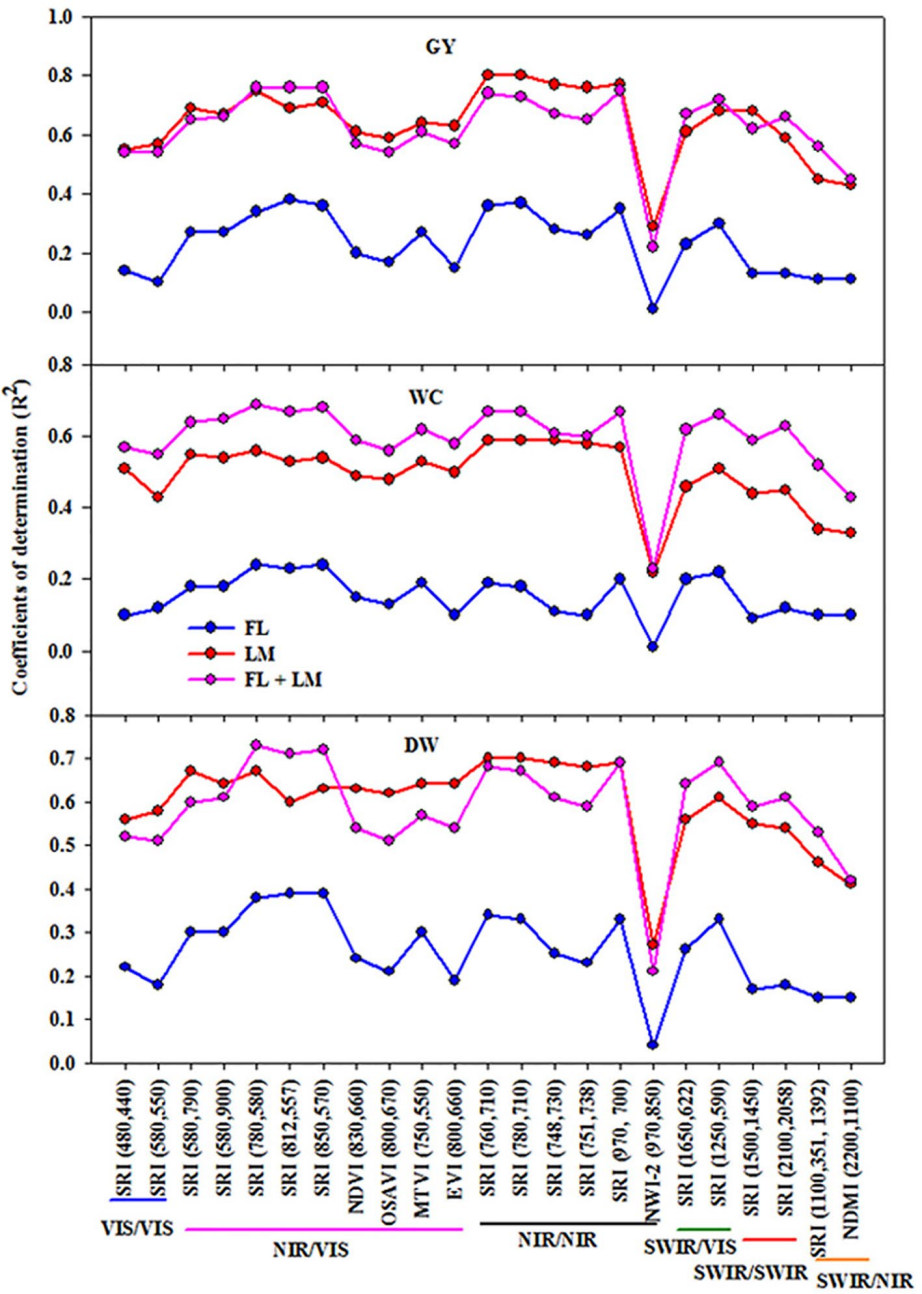
Coefficient of determinations (R^2^) for the linear relationships of different spectral reflectance indices with shoot dry weight per square meter (DW), water content of aboveground biomass (WC), and grain yield per hectare (GY) under full irrigation (FL), limited irrigation (LM) (n = 192), and the combined two treatments (FL+LM) (n = 384). R^2^ values ≥ 0.10 are significant at alpha = 0.05.

Twenty-three different SRIs (seven published indices and 16 indices constructed in this study from contour map analysis) were selected to cover different combinations of wavelengths from visible (VIS), near (NIR), and shortwave (SWIR) infrared and linearly regressed with the measured parameters under FL, LM, and FL+LM treatments (**Figure 2**). In general, all the SRIs correlated better with the measured parameters when they were calculated from the canopy spectral reflectance detected under the LM treatment compared with those detected under the FL treatment. When the data of canopy spectral reflectance of the FL and LM treatments were combined together, all the SRIs were efficiently correlated with the measured parameters as they did under the LM treatment (**Figure 2**).

The SRIs constructed in this study and based on NIR/VIS, NIR/NIR, and SWIR/VIS exhibited higher coefficients of determination (R^2^) with the measured parameters under FL, LM, and FL+LM treatments than those based on VIS/VIS, SWIR/SWIR, and SWIR/NIR. In addition, when the published SRIs based on NIR/VIS and NIR/NIR were compared with those constructed, the later SRIs exhibited higher values for R^2^ with the measured parameters than the former. The published normalized water index-2 (NWI-2 = (R_970_−R_850_)/(R_970_+R_850_)) was the only SRIs that failed to estimate the variation of the measured parameters under FL and attended a lower value of R^2^ under LM and FL+LM when compared to that of other SRIs (**Figure 2**).

### Phenotypic and Genotypic Correlations Between SRIs and Measured Parameters

**Figure 3:**
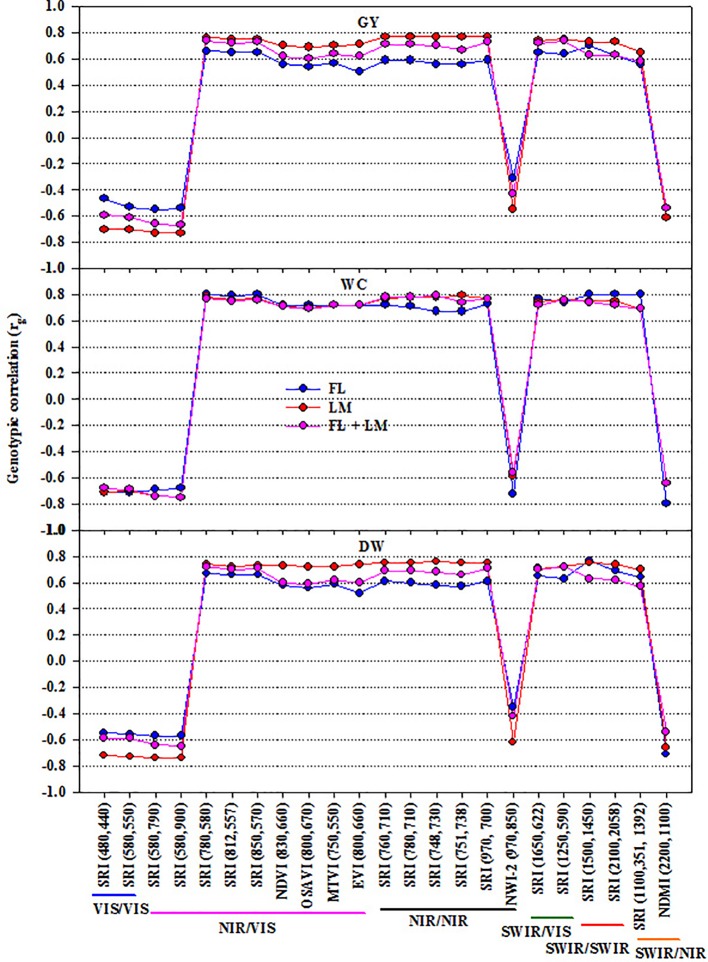
Genetic correlations between different spectral reflectance indices and measured parameters (shoot dry weight per square meter (DW), water content of aboveground biomass (WC), and grain yield per hectare (GY)) under full irrigation (FL), limited irrigation (LM), and the combined two treatments (FL+LM). R^2^ values ≥ 0.50 are significant at alpha = 0.05.

To illustrate the importance of SRIs as an indirect selection tool, the phenotypic and genetic correlation between SRIs and the three measured parameters were calculated (**Figure 3** and **Figure S3**). In general, all SRIs had significant positive and negative genetic correlations with the measured parameters under each treatment, except NWI-2 for DW and GY under FL and FL+LM (**Figure 3**). NWI-2 had a highly negative genetic correlation with DW under LM (–0.62) and WC under FL (–0.72). Most of the SRIs showed a strong genotypic correlation with WC under the three treatments (rg ≥ ± 0.70). The genotypic correlations between the SRIs and DW and GY were much stronger under LM than that under FL and FL+LM. Even though some SRIs exhibited a moderate relationship with the measured parameters, such as SRIs based on VIS/VIS and SWIR/SWIR, they showed a highly significant genetic correlation (rg ≥ ± 0.70) with DW and GY under LM and with WC under FL and LM (**Figure 3**).

### Broad-Sense Heritability of SRIs and Measured Parameters

**Figure 4:**
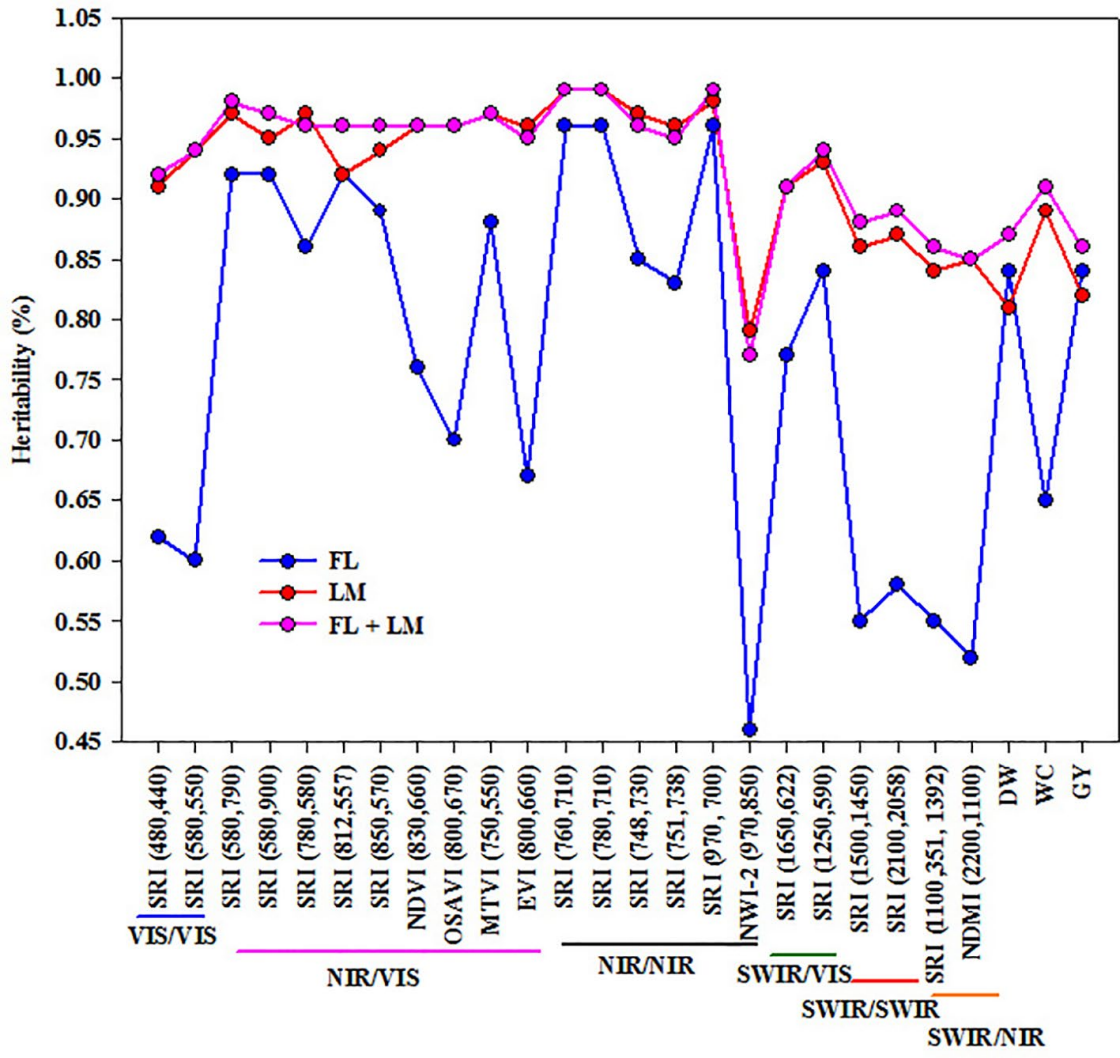
Broad-sense heritability (%) for different spectral reflectance indices and three measured parameters (shoot dry weight per square meter (DW), water content of aboveground biomass (WC), and grain yield per hectare (GY)) under full irrigation (FL), limited irrigation (LM), and the combined two treatments (FL+LM).

All the SRIs and the three measured parameters exhibited high heritability values under LM and FL+LM, with a range of 0.77 to 0.99 (**Figure 4**). Heritability was higher for most SRIs, especially those based on VIS/VIS, NIR/VIS, NIR/NIR, and SWIR/VIS than that of the three measured parameters under LM and FL+LM. Whereas the heritability value for SRIs based on SWIR/SWIR and SWIR/NIR was comparable with those three measured parameters under both conditions. SRIs based on VIS/VIS, SWIR/SWIR, and SWIR/NIR and NWI-2 resulted in lower heritability values than those of the three measured parameters under FL. Importantly, even though some SRIs exhibited low and moderate relationships with the measured parameters, they showed high heritability (**Figure 4**).

### Extraction of the Sensitive Spectral Band Intervals and Influential Wavelengths for Estimating the Measured Parameters Based on PLSR and SMLR Analysis

**Figure 5:**
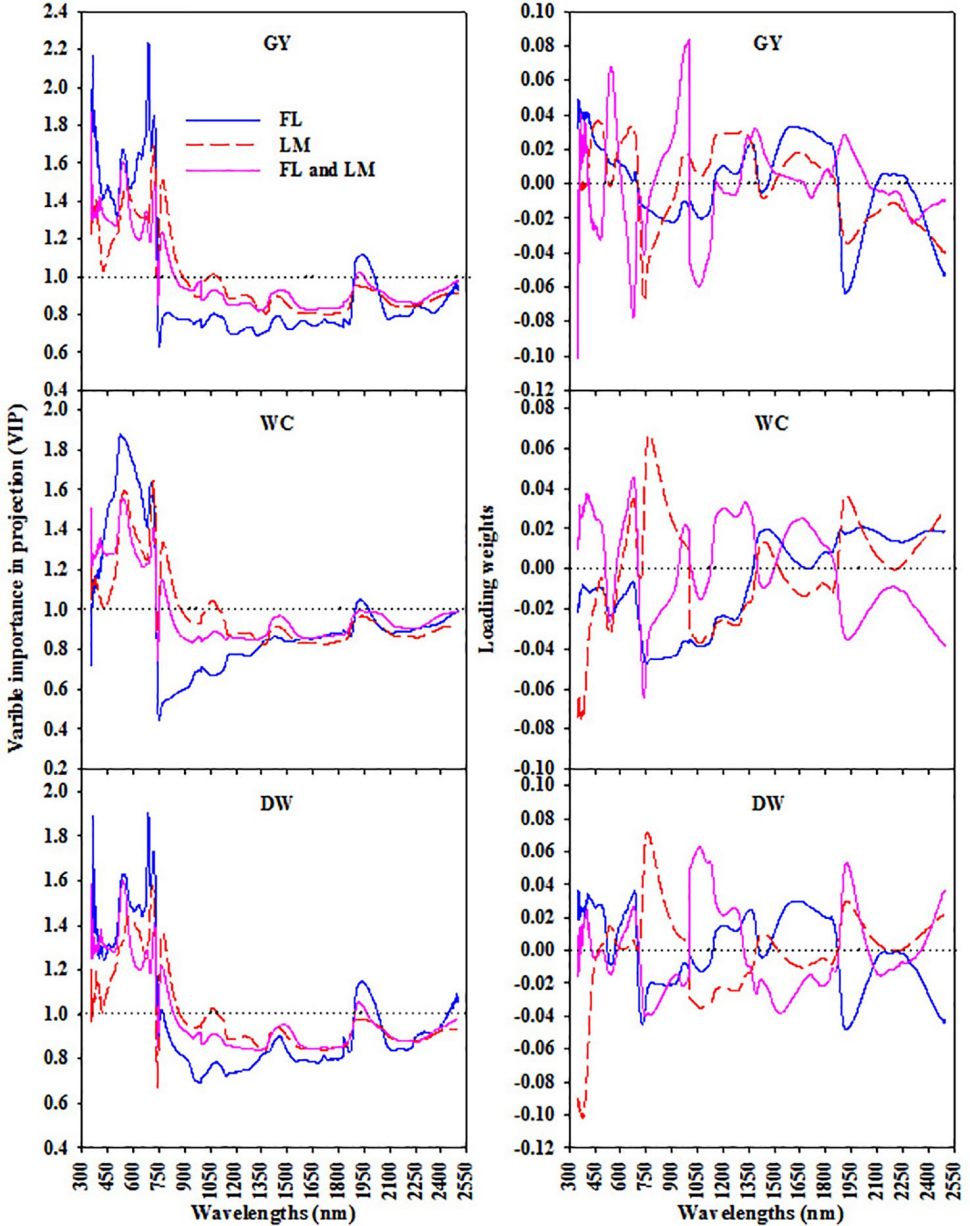
The variable importance in projection (VIP) and loading weights of PLSR analysis over full wavelengths to extract the sensitive spectral band intervals for each measured parameters [shoot dry weight per square meter (DW), water content of aboveground biomass (WC), and grain yield per hectare (GY)] under full irrigation (FL), limited irrigation (LM), and the combined two treatments (FL+LM).

Based on the variable importance in projection (VIP) and loading weights of the PLSR analysis over the full spectrum, the most sensitive spectral band intervals for each measured parameter under FL, LM, and FL+LM were extracted and listed in **Table 2**. These sensitive band intervals were extracted when their value of VIP was greater than 1.0 and was synchronized with a high absolute loading weight (**Figure 5**). Under the FL treatment, the VIS and red-edge band intervals, which were located at 350–774 nm for DW and 350–741 nm for GY, exhibited significant and weak relationships with DW, whereas they failed to exhibit any relationship with WC (**Table 2**). The SWIR band intervals that were extracted for the three parameters under FL (mainly 1,891–2,030 nm for DW, 1,899–1,978 nm for WC, and 1,891–2,010 nm for GY) exhibited significant and weak relationships with DW only, whereas they failed to exhibit any relationships with the other two parameters (**Table 2**). The wavelengths around 350–737 nm and 751–889 nm, which corresponded to VIS, red edge, and middle NIR band intervals, had a strong relationship with DW and GY under LM and FL+LM, and a weak relationship with WC under LM, whereas they failed to exhibit relationships with WC under FL+LM (**Table 2**). The SWIR band intervals that were extracted for WC and GY under LM treatment did not exhibit any relationship with either parameter, whereas those extracted under FL+LM had weak and significant relationship with DW and GY (**Table 2**).

**Table 2:** Extraction of the important sensitive spectral band intervals based on the variable importance in projection (VIP) and loading weights of partial least square regression (PLSR) analysis over full wavelengths as well as the most influential wavelengths and their models using the stepwise multiple linear regression (SMLR) for the three measured parameters [shoot dry weight per square meter (DW), water content of aboveground biomass (WC), and grain yield per hectare (GY)] under full irrigation (FL), limited irrigation (LM), and the combined two treatments (FL+LM).

Treatments	Par.	spectral band intervals	R^2^	RMSE	influential wavelength	Equation	Model R^2^	Model RMSE
**FL**	**DW**	350–774	0.34^*^	0.362	769	DW = 2.24 + 0.82 (R_769_) – 8.8 (R_1921_) – 2.1 (R_2443_)	0.19^**^	0.396
		1,891–2,030	0.18^*^	0.456	1,921			
		2,443–2,500	0.01	0.618	2,443			
	**WC**	359–733	0.06	5.183	733	WC = 88.82 – 18.58 (R_733_) – 50.99 (R_1899_)	0.12^*^	3.83
		1,899–1,978	0.01	10.57	1,899			
	**GY**	350–741	0.39^*^	0.937	693	GY = 9.38 – 14.74 (R_693_) – 8.69 (R_1891_)	0.23^**^	1.06
		1,891–2,010	0.07	1.247	1,891			
**LM**	**DW**	350–733	0.63^***^	0.166	733	DW = 1.09 – 5.56 (R_733_) + 4.99 (R_751_)	0.69^***^	0.147
		751–871	0.62^***^	0.167	751			
	**WC**	350–737	0.25^*^	4.640	532	WC = 63.44 – 74.6 (R_532_) + 41.7(R_751_) – 15.3 (R_1066_)	0.53^***^	3.17
		751–877	0.27^*^	4.976	751			
		1,026–1,098	0.001	6.985	1,066			
	**GY**	350–737	0.73^***^	0.468	737	GY = 2.87 – 31.4 (R_737_) + 30.8 (R_748_) – 1.2 (R_1061_)	0.77^***^	0.431
		748–889	0.73^***^	0.467	748			
		1,045–1,088	0.07	0.897	1,061			
**FL+LM**	**DW**	350–737	0.69^***^	0.309	737	DW = 1.35 –12.60 (R_737_) + 11.7 (R_750_) – 0.98 (R_1896_)	0.59^***^	0.351
		750–836	0.64^***^	0.329	750			
		1,896–1,968	0.38^*^	0.442	1,896			
	**WC**	350–734	0.23	5.189	557	WC = 67.15 – 95.5 (R_557_) + 27.9(R_812_)	0.65^***^	3.91
		753–812	0.05	5.717	812			
	**GY**	350–738	0.74^***^	0.952	738	GY = 3.63 – 46.8(R_738_) + 43.7 (R_751_) – 4.6 (R_1947_)	0.65^***^	1.12
		751–841	0.70^***^	1.032	751			
		1,903–1,947	0.28^*^	3.464	1,947			

*, **, *** Significant at the 0.05, 0.01, and 0.001 probability levels, respectively.

The sensitive band intervals that were extracted based on the VIP and loading weights of the PLSR analysis were further applied to SMLR analysis as independent variables to select the most influential wavelengths for each parameter under the three treatments (**Table 2**). In general, 23 individual wavelengths were identified as the most influential wavelengths for estimating the measured parameters. The most influential wavelengths for estimating the measured parameters were in the VIS region (693 nm), red-edge region (733 nm), middle NIR region (769 nm), and SWIR region (1,891, 1,899, 1,921, and 2,443 nm) under FL; in the VIS region (532 nm), red-edge region (733, 737, 748 nm), and NIR region (751, 1,061, and 1,066 nm) under LM; and in the VIS region (557 nm), red-edge region (737, 738, 750 nm), NIR region (751 and 812 nm), and the SWIR region (1,896 and 1,947 nm) under FL+LM (**Table 2**). Irrespective of the irrigation treatments, the most influential wavelengths for estimating DW were in the red-edge region (733, 737, and 750 nm), NIR region (751 and 769 nm), and SWIR region (1,896, 1,921, and 2,443 nm). The wavelengths identified for estimating WC were mainly in the VIS region (532 and 557 nm), red-edge region (733 nm), NIR region (751, 812, and 1,066 nm), and SWIR region (1,899 nm). For estimating GY, these wavelengths were mainly identified in the VIS region (693 nm), red-edge region (737, 738, and 748 nm), NIR region (751 and 1,061 nm), and SWIR region (1,891 and 1,947 nm) (**Table 2**).

### Calibration and Validation of PLSR Analysis for the Full Spectrum

To assess the measured parameters using the entire continuous spectrum, the PLSR analysis was estimated using the LOOCV and the ONLVs for avoiding overfitting of the spectral data. The ONLVs were identified based on the goodness of prediction accuracy (Q^2^) (**Figure S2**). The ONLVs were 6, 3, and 6 under FL; 5, 5, and 6 under LM; and 8, 6, and 9 under FL+LM for DW, WC, and GY, respectively (**Table 3**). In general, across all calibration (cal) and validation (val) data set formations, the closest relationship for the measured parameters were recorded under LM and FL+LM, with significant R^2^ cal or val > 0.70 for DW, >0.60 for WC, and >0.75 for GY (**Table 3**). The PLSR exhibited a moderate relationship in both cal and val for DW and GY (R^2^ ∼ 0.45) and a weak relationship for WC (R^2^ ∼ 0.20) under FL (**Table 3**). The lowest values for RMSE and relative error (RE) for the three measured parameters in either the cal or val set were recorded under LM. The RMSE in both sets under FL+LM was lower than those under FL, but the opposite was true for RE (**Table 3**).

**Table 3:** Calibration and validation statistics of partial least square regression (PLSR) models based on entire full wavelengths (350–2500 nm) for estimating shoot dry weight per square meter (DW), water content of aboveground biomass (WC), and grain yield per hectare (GY) under full irrigation (FL), limited irrigation (LM) (n = 192), and the combined two treatments (FL+LM) (n = 384). Twenty-five percent of data sets were applied for validation, while the remaining data sets were included in training set.

Treatments	Parameters	ONLVs	Calibration data set			Validation data set		
			R²	RMSE_._	RE (%)	R²	RMSE_._	RE (%)
**FL**	**DW**	6	0.44^**^	0.327	15.35	0.45^**^	0.333	15.63
	**WC**	3	0.19^*^	3.639	4.89	0.20^*^	3.658	4.78
	**GY**	6	0.43^**^	0.908	13.64	0.44^**^	0.902	13.55
**LM**	**DW**	5	0.71^***^	0.142	10.78	0.73^***^	0.137	10.42
	**WC**	5	0.61^***^	2.870	4.45	0.61^***^	2.840	4.40
	**GY**	6	0.79^***^	0.412	11.32	0.80^***^	0.401	11.01
**FL+LM**	**DW**	8	0.71^***^	0.295	17.08	0.70^***^	0.302	17.48
	**WC**	6	0.70^***^	3.586	5.16	0.70^***^	3.621	5.21
	**GY**	9	0.78^***^	0.872	16.95	0.77^***^	0.874	16.98

*, **, *** Significant at the 0.05, 0.01, and 0.001 probability levels, respectively.

ONLVs, optimal number of latent variables.

RE, relative error.

### Model Application for Estimating the Measured Parameters Based on Wavelengths and SRIs

SMLR analysis was performed to construct different models for the measured parameters based on the most influential wavelengths selected for each parameter under each treatment (**Table 2**) or the different groups of SRI that covered the VIS/VIS, NIR/VIS, NIR/NIR, SWIR/VIS, SWIR/SWIR, and SWIR/NIR (**Table 4**). In general, both models (wavelengths or different groups of SRI) delivered more accurate estimations of the measured parameters under LM and FL+LM than under FL. Under FL, the SRI models based on NIR/VIS, NIR/NIR, and SWIR/VIS were more accurate to estimate the measured parameters than was that of the other group of SRI models or wavelength models and explained 38% to 46%, 23% to 30%, and 35% to 46% of the variation in DW, WC, and GY, respectively (**Table 4**). The SRI models based on NIR/NIR under LM and NIR/VIS under FL+LM were the best models to accurately estimate the measured parameters, with the NIR/NIR model explaining 72%, 60%, and 80% and NIR/VIS model explaining 75%, 71%, and 78% of the variation in DW, WC, and GY respectively. In addition, the SRI models based on the NIR/VIS and wavelength models showed comparable accurate estimations of the measured parameters under LM and explained approximately 69%, 59%, and 77% of the variation in DW, WC, GY, respectively (**Tables 2** and **4**). Under FL+LM, the SRI models based on NIR/NIR and SWIR/VIS still had more accurate estimations of the measured parameters than did the wavelength models. The SRI models based on VIS/VIS, SWIR/SWIR, or SWIR/NIR provided the least accurate estimations of the measured parameters under the three treatments compared with that of the other models (**Tables 2** and **4**).

**Table 4:** SMLR Model summary for estimating the measured parameters based on different groups of SRIs.

SRIs	Treat.	Par.	Model R^2^	RMSE	Equation
SRI _(480,440)_**(1)** SRI _(580,550)_**(2)**	**FL**	**DW**	0.14^*^	0.384	**DW** = 8.36 – 4.69 **(1)** – 1.96 **(2)**
		**WC**	0.13^*^	3.81	**WC =** 111.2 – 16.4 **(1)** – 26.0 **(2)**
		**GY**	0.15^*^	1.12	**GY** = 20.5 – 11.2 **(1)** – 3.42 **(2)**
	**LM**	**DW**	0.61^***^	0.166	**DW** = 3.52 – 1.08 **(1)** – 1.13 **(2)**
		**WC**	0.51^***^	3.25	**WC** = 107.2 – 34.7 **(1)** – 5.3 **(2)**
		**GY**	0.60^***^	0.568	**GY** = 11.08 – 3.71 **(1)** – 3.72 **(2)**
	**FL+ LM**	**DW**	0.54^***^	0.369	**DW** = 6.24 – 2.85 **(1)** – 1.82 **(2)**
		**WC**	0.59^***^	4.24	**WC** = 128.6 – 40.5 **(1)** – 19.8 **(2)**
		**GY**	0.56^***^	1.22	**GY** = 20.6 – 9.52 **(1)** – 6.53 **(2)**
SRI _(580,790)_**(3)** SRI _(580,900)_**(4)** SRI _(780,580)_**(5)** SRI _(812,557)_**(6)** SRI _(850,570)_**(7)** NDVI _(830,660)_**(8)** OSAVI _(800,670)_**(9)** MTVI _(750,550)_**(10)** EVI _(800,660)_**(11)**	**FL**	**DW**	0.46^**^	0.330	**DW** = – 3.8 – 54.4**(3)** + 65.8**(4)** – 0.81**(5)** – 0.41**(6)** + 1.9 **(7)** + 6.1**(8)** – 6.6**(9)** + 0.001**(10)** + 0.85**(11)**
		**WC**	0.30^**^	3.49	**WC** = 56.9 – 155.6**(3)** + 256.2**(4)** – 0.66**(5)** – 4.6**(6)** + 9.1**(7)** – 70.2**(8)** + 94.3**(9)** + 0.53**(10)** – 51.9**(11)**
		**GY**	0.46^**^	0.903	**GY** = – 38.1 – 55.8**(3)** + 123.5**(4)** – 2.3**(5)** + 5.0**(6)** + 0.63**(7)** + 97.2**(8)** – 101.7**(9)** – 0.04**(10)** + 26.1**(11**)
	**LM**	**DW**	0.69^***^	0.150	**DW** = 0.52 – 2.7**(3)** + 2.5**(4)** + 0.07**(5)** + 0.01**(6)** + 0.11**(7)** – 1.7**(8)** + 2.5**(9)** – 0.002**(10)** – 0.58**(11)**
		**WC**	0.59^***^	3.02	**WC** = 36.7 + 0.57**(3)** + 0.61**(4)** + 2.5**(5)** + 12.4**(6)** – 10.6**(7)** + 39.5**(8)** – 85.4**(9)** + 0.32**(10)** + 46.7**(11)**
		**GY**	0.77^***^	0.441	**GY** = 4.3 – 7.1**(3)** – 1.01**(4)** + 1.6**(5)** + 0.84**(6)** – 1.5**(7)** – 18.3**(8)** + 22.8**(9)** – 0.02**(10)** – 7.0**(11)**
	**FL+ LM**	**DW**	0.75^***^	0.278	**DW** = –2.2 – 1.2**(3)** + 5.3**(4)** + 0.48**(5)** + 0.90**(6)** – 0.79**(7)** + 0.35**(8)** + 2.0**(9)** + 0.002**(10)** – 1.4**(11)**
		**WC**	0.71^***^	3.60	**WC** = 32.0 – 8.2**(3)** + 60.0**(4)** + 6.4**(5)** + 13.3**(6)** – 14.9**(7)** + 80.7**(8)** – 92.3**(9)** + 0.19**(10)** + 21.3**(11)**
		**GY**	0.78^***^	0.869	**GY** = – 14.2 – 7.3**(3)** + 21.9**(4)** + 1.4**(5)** + 5.7**(6)** – 4.8**(7)** + 22.9**(8)** – 15.7**(9)** – 0.04**(10)** + 2.6**(11)**
SRI _(760,710)_**(12)** SRI _(780,710)_**(13)** SRI _(748,730)_**(14)** SRI _(751,738)_**(15)** SRI _(970,700)_**(16)** NWI-2 _(970,850)_**(17)**	**FL**	**DW**	0.42^**^	0.338	**DW** = 1.3 + 2.5**(12)** – 2.4**(13)** + 22.6**(14)** – 24.0**(15)** + 0.30**(16)** – 9.0**(17)**
		**WC**	0.26^**^	3.55	**WC** = 115.4 + 7.6**(12)** – 6.2**(13)** + 335.3**(14)** – 391.8**(15)** + 2.6**(16)** – 46.8**(17)**
		**GY**	0.40^**^	0.950	**GY** = 22.5 – 6.3**(12)** + 8.5**(13)** + 68.8**(14)** – 91.2**(15)** + 0.21**(16)** – 18.1**(17)**
	**LM**	**DW**	0.72^***^	0.142	**DW** = – 3.7 + 0.96**(12)** – 1.7**(13)** + 16.7**(14)** – 11.9**(15)** + 0.36**(16)** – 1.2**(17)**
		**WC**	0.60^***^	2.98	**WC** = 1.4 + 11.3**(12)** – 12.3**(13)** + 33.6**(14)** + 20.0**(15)** + 2.0**(16)** – 9.8**(17)**
		**GY**	0.80^***^	0.404	**GY** = – 3.0 + 5.2**(12)** –2.5**(13)** – 58.9**(14)** + 61.5**(15)** – 0.26**(16)** – 1.7**(17)**
	**FL+ LM**	**DW**	0.71^***^	0.297	**DW** = 2.1 + 3.1**(12)** – 2.7**(13)** – 13.1**(14)** + 11.2**(15)** + 0.39**(16)** – 0.81**(17)**
		**WC**	0.70^***^	3.64	**WC** = 57.7 + 23.1**(12)** – 30.5**(13)** + 475.1**(14)** – 475.7**(15)** + 6.2**(16)** + 0.87**(17)**
		**GY**	0.76^***^	0.912	**GY** = 15.2 + 5.7**(12)** – 2.8**(13)** – 16.1**(14)** – 0.94**(15)** + 0.75**(16)** + 1.1**(17)**
SRI _(1650,622)_**(18)** SRI _(1250,590)_**(19)**	**FL**	**DW**	0.38^**^	0.346	**DW** = 0.73 – 0.53 **(18)** + 0.64 **(19)**
		**WC**	0.23^**^	3.59	**WC =** 64.2 – 0.91 **(18)** + 2.52 **(19)**
		**GY**	0.35^**^	0.974	**GY** = 2.95 – 1.44 **(18)** + 1.73 **(19)**
	**LM**	**DW**	0.62^***^	0.164	**DW** = 0.52 – 0.18 **(18)** + 0.36 **(19)**
		**WC**	0.52^***^	3.20	**WC =** 51.5 – 4.01 **(18)** + 6.75 **(19)**
		**GY**	0.69^***^	0.496	**GY** = 0.74 – 0.86 **(18)** + 1.47 **(19)**
	**FL+ LM**	**DW**	0.71^***^	0.292	**DW** = 0.25 – 0.49 **(18)** + 0.69 **(19)**
		**WC**	0.66^***^	3.85	**WC =** 52.3 – 3.18 **(18)** + 6.18 **(19)**
		**GY**	0.74^***^	0.936	**GY** = 0.04 – 1.66 **(18)** + 2.35 **(19)**
SRI _(1500,1450)_**(20)** SRI _(2100,2058)_**(21)**	**FL**	**DW**	0.19^**^	0.397	**DW** = – 4.03 + 1.82 **(20)** + 3.08 **(21)**
		**WC**	0.13^*^	3.82	**WC =** 33.6 – 22.1 **(20)** + 56.2 **(21)**
		**GY**	0.13^*^	1.13	**GY** = – 7.9 + 5.85 **(20)** + 5.62 **(21)**
	**LM**	**DW**	0.55^***^	0.178	**DW** = – 2.57 + 2.68 **(20)** + 0.60 **(21)**
		**WC**	0.45^**^	3.44	**WC =** 2.24 + 8.38 **(20)** + 46.1 **(21)**
		**GY**	0.60^***^	0.570	**GY** = – 10.18 + 3.12 **(20)** + 8.90 **(21)**
	**FL+ LM**	**DW**	0.61^***^	0.339	**DW** = – 5.3 – 0.62 **(20)** + 6.58 **(21)**
		**WC**	0.64^***^	4.0	**WC =** – 15.3 – 26.1 **(20)** + 99.1 **(21)**
		**GY**	0.66^***^	1.08	**GY** = – 19.3 – 4.43 **(20)** + 25.3 **(21)**
SRI _(1100,351,1392)_**(22)** NDMI _(2200,1100)_**(23)**	**FL**	**DW**	0.16^*^	0.404	**DW** = 0.36 + 2.26 **(22)** – 2.03 **(23)**
		**WC**	0.05^*^	3.99	**WC =** 68.55 + 23.4 **(22)** + 1.04 **(23)**
		**GY**	0.11^*^	1.14	**GY** = 2.36 + 4.73 **(22)** – 5.31 **(23)**
	**LM**	**DW**	0.46^**^	0.194	**DW** = 0.72 + 2.46 **(22)** – 0.31 **(23)**
		**WC**	0.35^**^	3.73	**WC =** 53.5 + 26.9 **(22)** – 12.8 **(23)**
		**GY**	0.46^**^	0.659	**GY** = 1.32 + 6.58 **(22)** – 2.33 **(23)**
	**FL+ LM**	**DW**	0.54^***^	0.370	**DW** = 0.85 + 7.40 **(22)** + 1.60 **(23)**
		**WC**	0.52^***^	4.58	**WC =** 56.9 + 80.3 **(22)** + 11.51 **(23)**
		**GY**	0.57^***^	1.22	**GY** = 2.07 + 25.5 **(22)** + 5.4 1 **(23)**

*, **, *** Significant at the 0.05, 0.01 and 0.001 probability levels, respectively.

FL, LM, and FL+LM, full irrigation, limited irrigation, and the combined two treatments.

DW, WC, and GY, shoot dry weight per square meter, water content of aboveground biomass, and grain yield per hectare.

### Prediction of Measured Parameters Based on Influential Wavelengths and SRIs Using the PLSR Model

**Figure 6:**
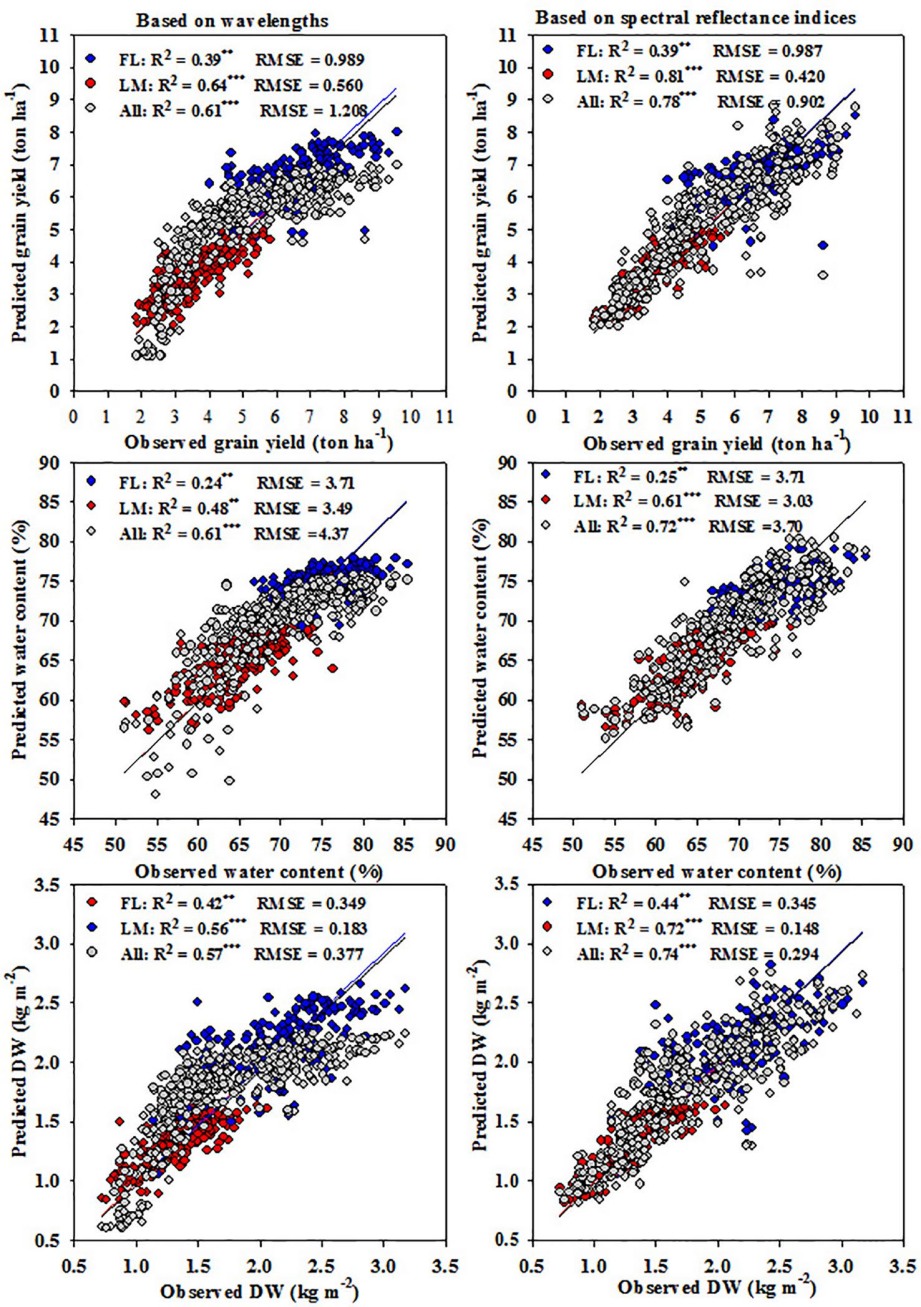
Relationship between estimated and observed measured parameters ((shoot dry weight per square meter (DW), water content of aboveground biomass (WC), and grain yield per hectare (GY)) under full irrigation (FL), limited irrigation (LM), and the combined two treatments (All) based on influential wavelengths or SRIs.

The PLSR models using the influential wavelengths selected for the three parameters under each treatment, which were 7, 8, and 8 wavelengths under FL, LM, and FL+LM, respectively, or all twenty-three SRIs were established to predict the three measured parameters under each treatment, and scatter plots and linear regression between the observed and predicted values of each measured parameter are shown in **Figure 6**. In general, the PLSR models based on the SRIs exhibited higher values of R^2^ and lower values of RMSE between observed and predicted values of the measured parameters than did those of the PLSR models based on wavelengths. Both models fitted the three measured parameters more precise under the LM (R^2^ values ranged from 0.48 to 0.64 for wavelength models and from 0.61 to 0.81 for SRI models) and FL+LM (R^2^ values ranged from 0.57 to 0.61 for wavelength models and from 0.72 to 0.78 for SRI models) than under the FL (R^2^ values ranged from 0.16 to 0. 31 for wavelength models and from 0.25 to 0.44 for SRI models) (**Figure 6**). Interestingly, the PLSR model based on all SRIs exhibited comparable performance for estimating the measured parameters (**Figure 6**) as did the SMLR model that was based on NIR/VIS, NIR/NIR, or SWIR/VIS (**Table 4**) and the individual SRIs that were selected within NIR/VIS, NIR/NIR, or SWIR/VIS under each treatment (**Figure 2**). However, the SMLR model based on the influential wavelengths performed better for the three measured parameters (**Table 2**) than the wavelength models derived by PLSR (**Figure 6**), especially under LM (R^2^ values ranged from 0.56 to 0.77 for SMLR model and from 0.59 to 0.65 for the PLSR model) and FL+LM (R^2^ values ranged from 0.59 to 0.65 for SMLR model and from 0.57 to 0.61 for PLSR model).

## Discussion

The simultaneous indirect assessment of a number of different destructive parameters in a rapid and cost-efficient way by incorporating nondestructive tools into genetic drought studies will become increasingly important for increasing the chances of recognizing the genotypes that are well-adapted to water shortage in arid conditions. Interestingly, using a nondestructive tool, such as a proximal canopy spectral reflectance, is not only useful for achieving the previous objective but also useful for creating indirect nondestructive traits that can be exploited as an alternative to the direct traits (measured traits) in breeding trials. However, these indirect traits will be appropriate as screening criteria if such traits have a strong genetic correlation and have a high heritability as the direct traits (Babar et al., 2006; Gutierrez et al., 2010). To obtain accurate information from canopy spectral reflectance to assess the direct traits or create the indirect traits, further studies are needed to fix many factors associated with this tool as a methodology and the condition of the reflectance measurements, equipment used, plant materials evaluated, and analysis of spectral data before being applied in breeding trials (Lobos et al., 2019).

In the present study, a sufficient number of RILs were evaluated under two contrasting irrigation regimes (FL and LM) for 2 years to create a wide range in the investigated crop variables, which is an important and first step to make spectral reflectance measurements as realistic as possible for plant breeding programs. The ability of different constructed and published spectral reflectance indices (SRIs) for assessing the destructive parameters were evaluated through linear regression analysis, genotypic correlations, and heritability. As the SRIs involved only 2–3 wavelengths and targeted only the spectral information over a wide range waveband (Hansen and Schjoerring, 2003; Li et al., 2014; Stellacci et al., 2016), different multivariate analysis (PLSR and SMLR) were further applied to extract the sensitive spectral band intervals and wavelengths associated with growth, yield, and plant water status from the full spectrum (350–2500 nm). This multivariate analysis revealed important spectral information correlated with crop parameters across a broad range of crop growing conditions.

Interestingly, the results of this study indicate that all SRIs had weaker relationships with the three detective parameters under the full irrigation treatment (FL), whereas they had a better performance under the limited irrigation treatment (LM) or when the SRIs were combined across the two treatments (FL+LM) (**Figure 2**). This finding reflects that, methodologically, crop growing conditions play vital roles in the values of canopy spectral reflectance. This finding could be attributed to the fact that large leaf area index and biomass saturation under FL and high temperature under arid conditions could make the SRIs unsuitable to differentiate genotypic differences in plant growth, water status, and yield, especially at the early growth stage. These results also suggest that sufficient genetic variation in morphological characteristics must exist when measuring the spectral reflectance. In this study, the drought-tolerant genotype (Sakha 94) and drought-susceptible genotype (Sids 1) were grouped together in the same cluster under FL, whereas they were separated in two different clusters under LM and FL+LM based on the three measured parameters (**Figure 1** and **Table 1**). This indicates that the both parents have a low morphological diversity and had the lowest range in the measured parameters under FL (**Table 1**), which could explain why the association between all the SRIs and three detective parameters was generally low under FL, when compared to LM condition. Previous studies have reported that several SRIs exhibit a low association with several productive and physiological traits at the early stage (for example, at the booting stage in wheat) under full irrigation conditions, and these associations were improved when SRI data from the contrasting irrigation regimes (full irrigation, and mild and severe water deficit) were combined (Aparicio et al., 2000; Gutierrez et al., 2010; Lobos et al., 2014; El-Hendawy et al., 2017a; Garriga et al., 2017).

To exploit the SRIs as indirect selection tool in breeding trials, these indices should show high genetic correlation with and higher heritability than the direct destructive traits (Jackson, 2001; Gutierrez et al., 2010). The results of the present study showed that even though most of the SRIs exhibited low relationships with the measured parameters under the FL treatment (**Figure 2**), they could be used for assessing the direct traits because they have moderate and high genetic correlations and exhibited high heritability (**Figures 3** and **4**), especially the SRIs based on NIR/VIS, NIR/NIR, and SWIR/VIS wavelengths. These indices exhibited a better fit with the measured parameters under LM and FL+LM, as well as giving high genetic correlations, especially under LM, and exhibited higher heritability than the measured parameters under all conditions (**Figures 2**–**4**). These results suggest that because the SRIs based on NIR/VIS, NIR/NIR, and SWIR/VIS wavelengths demonstrated a high genetic base (high heritability and genetic correlation), these indices could be used for breeding purposes as indirect selection tools under both FL and LM treatments. A high heritability value for these indices indicates that the variation which is observed in the tested materials is mostly related to genotypic variation among the RILs rather than the environmental changes. A similar result was reported by Gutierrez et al. (2010) for assessing yield in elite wheat genotypes under two contrasting water stress conditions (well-watered and water-deficit conditions), who reported that the SRIs, especially those showing strong genetic correlations and reasonably high heritability, could be used for breeding purposes for high yielding advanced lines under both conditions.

### Integration Between PLSR and SMLR for Dealing With the Entire Spectral Data Set

Even though the SRIs represent a very simple approach for estimating the direct parameters and can be used as an alternative screening tool in plant breeding trials and to develop lightweight spectral sensors, many limitations are associated with this model to differentiate genotypes, such as the sensitivity of many bands involved in the SRIs for different physical and biochemical attributes of the canopy rather than the target traits (Ollinger, 2011). Therefore, recent studies have considered the entire spectral data set for improving the estimation of measured parameters using multivariate analysis. The advantage of this approach is the ability to predict crop variables under complex conditions, e.g., different agronomic treatments, heterogeneous field conditions, and different growth stages and genotypes (Darvishzadeh et al., 2008; Li et al., 2014; El-Hendawy et al., 2019a). In this study, the integration between PLSR and SMLR was applied to select the sensitive band intervals and influential wavelengths associated with each parameter under FL, LM, and FL+LM (**Table 2**). The sensitive band intervals were selected based on the values of VIP and absolute loading weight derived from PLSR analysis (**Figure 5**). Overall, the VIS (350–700), red-edge (700–750), and middle NIR (750–890) band intervals were identified as the most important regions for estimation of the measured parameters. These regions exhibited moderate relationships with DW and GY under FL and strong relationships with both parameters under LM and FL+LM, whereas they failed to track changes in WC under FL and FL+LM; while showing moderate relationships with WC under LM (**Table 2**). Indeed, the VIS region is known to be related to the pigment status and photosynthetic capacity, which always are the elements most affecting the growth and production of crops (Weber et al., 2012; Wang et al., 2017). The red-edge and NIR regions are mainly influenced by the structural leaf compounds, leaf cellular structure, and canopy structure, and therefore both regions are more informative for above ground biomass estimation than the other regions (Rotbart et al., 2013; Barankova et al., 2016; Wang et al., 2017). This influence may explain why these three regions were associated with DW and GY under all conditions in the present study. The water absorption bands in the NIR region, which penetrate deeper into the canopy, are also correlated well with leaf moisture content (Babar et al., 2006; Gutierrez et al., 2010). This correlation could explain why a relationship exists between the NIR region and WC under the LM treatment only.

When the sensitive band intervals selected by PLSR were applied to SMLR analysis as independent variables, different wavelengths were identified as the most effective wavelengths for monitoring the three measured parameters (**Table 2**). These influential wavelengths correspond to the visible green region (532 and 557 nm), middle of the red depression region (693 nm), red-edge inflection point (733, 737, 738, 748, and 750 nm), NIR region (751, 769, 814, 1,061, and 1,066 nm), and SWIR region (1,891, 1,896, 1,899, 1,921, 1,947, and 2,443 nm). The green region around 550 nm was found to be highly correlated with the high concentrations of chlorophyll *a* and *b* and directly related to photosynthetic efficiency (Christenson et al., 2016). Therefore, the wavelengths around 550 nm, such as 559 nm, have been used alone as well as in a ratio with some wavelengths in the red region to explain 13% to 92% of the variation in yield of wheat and soybean genotypes (Ma et al., 2001; Royo et al., 2003). In the present study, the two green wavelengths were extracted as the influential wavelengths for WC under LM and FL+LM (**Table 2**). This result indicates that the variability in WC among genotypes under water deficit stress could also be detected by the spectral properties related to pigment concentrations. Similar results have been reported by El-Hendawy et al. (2019a) and Kovar et al. (2019), who showed that the green spectral regions had a significant relationship with plant water status, as expressed by the relative water content, leaf water potential, and equivalent water thickness in wheat and soybean under different irrigation regimes. This could be explained by the loss of cell turgor under water stress leading to a decrease in cell volume (shrinking of cells), which ultimately results in a significant reduction in chlorophyll content, and therefore, high reflection in the spectral green region (Canny and Huang, 2006; Scoffoni et al., 2014).

Unlike the green region, the wavelengths in the red region are associated with the chlorophyll absorption capacity (Christenson et al., 2016). In the present study, the wavelength of 693 nm was important for estimating GY under FL (**Table 2**). This indicates that the high-yielding genotypes under FL conditions had lower reflectance in the red region, suggesting a higher amount of chlorophyll, which resulted in higher grain yields. This finding is consistent with that of Royo et al. (2003) for wheat and Weber et al. (2012) for maize.

The red-edge region was often used as an indirect stress indicator, especially when the plants suffer stress. This region carries important information about biomass quantity and leaf area index, and therefore, could be used to distinguish plant health and yield (Smith et al., 2004; Gitelson et al., 2011; El-Hendawy et al., 2019b). The five wavelengths extracted within this region were associated with DW and GY under LM and FL+LM (**Table 2**). This indicates that these wavelengths could be used to differentiate genotypic differences in DW and GY under limited water conditions.

The wavelengths in the NIR region were mainly influenced by several leaf structure properties, such as area, biomass and anatomy of leaves, intercellular air spaces, the ratio between palisade and spongy mesophylls, and the arrangement of cells within the mesophyll layer, as well as the leaf water status (Gutierrez et al., 2010; Li et al., 2014; Wang et al., 2017). We assumed that the evaluation of an increased number of genotypes under different irrigation rates could result in a significant variation among genotypes in the previous leaf structure properties. This indicates that the three measured parameters could be estimated through several wavelengths in the NIR region. The wavelengths extracted in the NIR region in this study also fully confirmed this statement and were informative regarding DW under FL and LM, and GY and WC under LM and FL+LM (**Table 2**).

The wavelengths in the SWIR region are always sensitive to plant water status and less sensitive to noises caused by the internal leaf structure (Mariotto et al., 2013; Rapaport et al., 2017). In addition, the spectral reflectance in this region was also found to be affected by leaf biochemical compounds, such as lignin, cellulose, sugar, proteins, and lipids (Romero et al., 2012; Wang et al., 2013). Yao et al. (2014) reported that the wavelength of 1865 nm was found to be sensitive to cellulose in wheat under different water and nitrogen treatments. Romero et al. (2012) also reported that because cellulose and lignin are the major components of plant dry matter, several wavelengths in the SWIR region (2100 to 2500 nm) are effective to correlate with plant dry matter content. In the present study, the five wavelengths extracted in SWIR were informative for the three parameters under FL conditions, as well as for DW and GY under FL+LM (**Table 2**). Interestingly, no wavelengths extracted in the SWIR region were informative for any of the three parameters under the LM conditions (**Table 2**). This could be because the leaf biochemical compounds may be affected at the same rate for all genotypes under LM conditions. Therefore, the wavelengths in the VIS and red edge, as well as the wavelengths related to water bands in the NIR region were sufficient to detect the changes in DW, GY, and WC under LM conditions in this study.

### Best Models for Estimating the Measured Parameters

Several studies have reported that constructing different models of the spectral reflectance data could improve the estimation potential of the measured parameters (Li et al., 2014; Christenson et al., 2016; Garriga et al., 2017; Lobos et al., 2019). In the present study, different models were constructed using SMLR and PLSR analysis. Some models were constructed based on the most influential wavelengths, which were selected for each parameter under each condition (**Table 2**) or based on different groups of SRIs (**Table 4**) using SMLR analysis, whereas other models were constructed based on all the most influential wavelengths, different SRIs groups (**Figure 6**), or the entire full spectrum (**Table 3**) using PLSR analysis. By modeling the spectral reflectance, an assessment of the important direct traits or the creation of new indirect ones is possible and can be applied in wheat breeding programs oriented toward adaptation to challenging water deficits in arid conditions. In this study, among all the models, there were five models for each condition (FL, LM, or FL+LM) that were more accurate in the estimation of the measured parameters than the other models. The SMLR models based on SRIs of NIR/VIS or NIR/NIR, as well as the PLSR models based on all SRIs or the full wavelengths were shared for the three conditions in addition to the PLSR and SMLR models based on most influential wavelengths for FL and LM, respectively, and the SMLR based on SRIs of SWIR/VIS for FL+LM. The five models identified for each condition explained 42% to 46%, 19% to 30%, and 39% to 46% under FL; 69% to 72%, 59% to 61%, and 77% to 81% under LM; and 71% to 75%, 61% to 71%, and 74% to 78% under FL+LM of the variation in DW, WC, and GY among genotypes, respectively (compared data in **Tables 2**–**4** and **Figure 6**). Interestingly, there were specific individual SRIs, especially those developed in this study and based on NIR/VIS or NIR/NIR, had a comparable performance for estimating the measured parameters, as for the previous five models (**Figure 2**). Taken together, these results indicate that it is possible to use the SRIs as a simple and easy way to estimate the growth, yield, and water status of wheat in breeding programs, especially those showing strong genetic correlations and reasonably high heritability. More importantly, the most efficient indices, which had significant relationships with the measured parameters, were based on VIS and NIR wavebands, which support ongoing efforts to develop new spectral sensors being less expensive and enabling more multilateral applications. However, the main drawback of SRIs is that it is difficult to create a universal index to remotely estimate crop variables for different genotypes. This is because the canopy reflectance is strongly influenced by the variation in structural and biochemical properties of the canopy among genotypes (Thenkabail et al., 2000; Li et al., 2014). For instance, although the different normalized water index (NWIs) using the wavelengths 970, 920, and 850 nm were the only indices satisfactorily explaining a large amount of variation in GY among wheat genotypes under full-irrigated and water-stressed conditions and added genotypic variation explanation to the studies of Prasad et al. (2007) and Gutierrez et al. (2010), the NWI-2 exhibited weak relationships with the measured parameters under all conditions in this study (**Figure 2**). In addition, even though the different normalized vegetative indices (NDVIs) have provided a strong association with wheat GY under different water stress conditions in the studies by Raun et al. (2001) and Royo et al. (2003), these indices failed to estimate GY in bread wheat in the study by Gutierrez et al. (2010) and exhibited low to moderate association with the measured parameters in the present study. Because of this inconsistency of SRIs in their relationship with the crop variables, multivariate analyses have been proposed and applied in recent decades for trait modeling (Nguyen and Lee, 2006; Garriga et al., 2017; Kawamura et al., 2018; Li et al., 2018; El-Hendawy et al., 2019a; Lobos et al., 2019). Among multivariate analysis, SMLR and PLSR methods have been widely used for estimating crop variables. In most studies, PLSR was superior to SMLR for trait modeling. However, to improve the performance of SMLR models, it is important to apply the model after selection of appropriate wavelengths (Goicoechea and Olivieri, 2002; Li et al., 2016; Kawamura et al., 2018). In the present study, there were three SMLR models, which are based on SRIs of NIR/VIS or NIR/NIR for all conditions and based on most the influential wavelengths for the LM condition, providing accurate estimation for the three measured parameters, similar to the PLSR models.

## Conclusion

The results of this study showed that the three destructive measurements (DW, WC, and GY) could be used as direct traits in breeding programs to assess the water stress tolerance among several advanced lines of spring wheat. However, there is a pressing need to assess these traits in a fast and nondestructive way to accelerate the development of genotypes for water stress conditions. In this study, the feasibility of applying spectral reflectance data for indirect assessment of these traits or exploited as an alternative to indirect traits was evaluated. The results indicated that it was possible to assess direct traits using the specific individual SRIs, especially those developed in this study and based on NIR/VIS wavelengths such as SRI _(580,790)_, SRI _(580,900)_, SRI _(780,580)_, SRI _(812,557)_, and SRI _(850,570)_ or NIR/NIR wavelengths such as SRI _(760,710)_, SRI _(780,710)_, SRI _(748,730)_, SRI _(751,738)_, and SRI _(970,700)_. Such SRIs could also be used as indirect traits for breeding purposes because they demonstrated high heritability and genetic correlation. Because there was no universal SRI that could be used to assess the direct traits under contrasting environmental conditions and with different genotypes, the methodology used in the selection of the important spectral waveband regions and wavelengths is becoming important. In this study, the integration between PLSR and SMLR was used to consider the entire spectral data set to improve the estimation of direct traits. The results confirmed that modeling of the spectral reflectance data using both analyses aided in constructing a robust model to assess some key breeding traits for breeding purposes of spring wheat genotypes under different environmental conditions.

## Data Availability Statement

All datasets generated for this study are included in the article/**Supplementary Material**.

## Author Contributions

SE-H performed the experiments and edited the manuscript, MA, NA, WH, YD, and KG designed the experiment, and followed upon data collection, SE-H, NA, ME, SE analysed the data, SE-H, MA, NA, KG, and WH Canopy spectral reflectance measurements, all authors helped in the interpretation of results, and read and approved the final manuscript.

## Funding

This work was supported by the Deanship of Scientific Research at King Saud University for Research Group No (RG-1435-032).

## Conflict of Interest

The authors declare that the research was conducted in the absence of any commercial or financial relationships that could be construed as a potential conflict of interest.

The handling editor is currently organizing a Research Topic with one of the authors [US], and confirms the absence of any other collaboration.
