# Supplementary material for: Comparative Performance of Spectral Reflectance Indices and Multivariate Modeling for Assessing Agronomic Parameters in Advanced Spring Wheat Lines Under Two Contrasting Irrigation Regimes
Source: Front Plant Sci. 2019 Nov 28;10:1537. doi: 10.3389/fpls.2019.01537 (PMC6892836; doi:10.3389/fpls.2019.01537)
Supplement: Supplementary file 1 [file Table_1.docx]

**Table S1. Genotypic variance (σ^2^_G_), environmental variance (σ^2^_e_), and phenotypic variance (σ^2^_P_) across years for different spectral reflectance indices and three agronomic parameters shoot dry weight per square meter (DW), water content of aboveground biomass (WC), and grain yield per hectare (GY)) under full irrigation (FL), limited irrigation (LM), and the combined two treatments (FL+LM).**

| **Parameters** | **FI** | | | **LM** | | | **FI+LM** | | |
| --- | --- | --- | --- | --- | --- | --- | --- | --- | --- |
|  | **σ^2^_G_** | **σ^2^_e_** | **σ^2^_P_** | **σ^2^_G_** | **σ^2^_e_** | **σ^2^_P_** | **σ^2^_G_** | **σ^2^_e_** | **σ^2^_P_** |
| **DW** | 0.1485 | 0.0283 | 0.1768 | 0.0534 | 0.0125 | 0.0659 | 0.1570 | 0.0235 | 0.1804 |
| **WC** | 6.6949 | 3.6050 | 10.2999 | 13.7213 | 1.6959 | 15.4172 | 14.5954 | 1.4435 | 16.0390 |
| **GY** | 1.0662 | 0.2031 | 1.2692 | 0.6911 | 0.1517 | 0.8428 | 1.4358 | 0.2337 | 1.6695 |
| **SRI _(480,440)_** | 0.0003 | 0.0002 | 0.0005 | 0.0053 | 0.0005 | 0.0058 | 0.0030 | 0.0003 | 0.0032 |
| **SRI _(580,550)_** | 0.0000 | 0.0000 | 0.0001 | 0.0109 | 0.0007 | 0.0116 | 0.0059 | 0.0004 | 0.0063 |
| **SRI _(580,790)_** | 0.0009 | 0.0001 | 0.0010 | 0.0076 | 0.0002 | 0.0078 | 0.0045 | 0.0001 | 0.0045 |
| **SRI _(580,900)_** | 0.0008 | 0.0001 | 0.0009 | 0.0054 | 0.0003 | 0.0056 | 0.0033 | 0.0001 | 0.0034 |
| **SRI _(780,580)_** | 0.7790 | 0.1268 | 0.9058 | 0.9763 | 0.0302 | 1.0065 | 1.1347 | 0.0473 | 1.1820 |
| **SRI _(812,557)_** | 0.3914 | 0.0340 | 0.4254 | 0.3953 | 0.0344 | 0.4296 | 0.4895 | 0.0204 | 0.5099 |
| **SRI _(850,570)_** | 0.5854 | 0.0724 | 0.6578 | 0.6190 | 0.0395 | 0.6585 | 0.7609 | 0.0317 | 0.7926 |
| **NDVI _(830,660)_** | 0.0009 | 0.0003 | 0.0012 | 0.0196 | 0.0008 | 0.0204 | 0.0104 | 0.0004 | 0.0108 |
| **OSAVI _(800,670)_** | 0.0006 | 0.0003 | 0.0009 | 0.0198 | 0.0008 | 0.0207 | 0.0102 | 0.0004 | 0.0106 |
| **MTVI _(750,550)_** | 24.4724 | 3.3371 | 27.8095 | 273.585 | 8.461 | 282.046 | 151.866 | 4.6969 | 156.563 |
| **EVI _(800,660)_** | 0.0011 | 0.0005 | 0.0017 | 0.0249 | 0.0010 | 0.0260 | 0.0135 | 0.0007 | 0.0142 |
| **SRI _(760,710)_** | 0.0856 | 0.0036 | 0.0892 | 0.2445 | 0.0025 | 0.2470 | 0.1931 | 0.0020 | 0.1950 |
| **SRI _(780,710)_** | 0.0948 | 0.0040 | 0.0988 | 0.2523 | 0.0025 | 0.2549 | 0.2031 | 0.0021 | 0.2052 |
| **SRI _(748,730)_** | 0.0005 | 0.0001 | 0.0006 | 0.0022 | 0.0001 | 0.0022 | 0.0016 | 0.0001 | 0.0016 |
| **SRI _(751,738)_** | 0.0004 | 0.0001 | 0.0005 | 0.0016 | 0.0001 | 0.0016 | 0.0012 | 0.0001 | 0.0012 |
| **SRI _(970, 700)_** | 0.3809 | 0.0159 | 0.3968 | 0.6945 | 0.0142 | 0.7087 | 0.6178 | 0.0062 | 0.6241 |
| **NWI-2 _(970,850)_** | 0.0000 | 0.0000 | 0.0000 | 0.0002 | 0.0001 | 0.0003 | 0.0001 | 0.0000 | 0.0001 |
| **SRI _(1650,622)_** | 0.3041 | 0.0908 | 0.3949 | 0.4235 | 0.0419 | 0.4654 | 0.4575 | 0.0452 | 0.5027 |
| **SRI _(1250,590)_** | 0.6413 | 0.1221 | 0.7634 | 0.7182 | 0.0541 | 0.7723 | 0.8496 | 0.0542 | 0.9038 |
| **SRI _(1500,1450)_** | 0.0003 | 0.0002 | 0.0005 | 0.0030 | 0.0005 | 0.0034 | 0.0019 | 0.0003 | 0.0022 |
| **SRI _(2100,2058)_** | 0.0004 | 0.0003 | 0.0007 | 0.0025 | 0.0004 | 0.0029 | 0.0019 | 0.0002 | 0.0021 |
| **SRI _(1100,351, 1392)_** | 0.0005 | 0.0004 | 0.0010 | 0.0029 | 0.0005 | 0.0034 | 0.0019 | 0.0003 | 0.0022 |
| **NDMI _(2200,1100)_** | 0.0005 | 0.0005 | 0.0010 | 0.0057 | 0.0010 | 0.0068 | 0.0031 | 0.0005 | 0.0037 |
